# Nesfatin‐1 inhibits myocardial ischaemia/reperfusion injury through activating Akt/ERK pathway‐dependent attenuation of endoplasmic reticulum stress

**DOI:** 10.1111/jcmm.16481

**Published:** 2021-05-03

**Authors:** Rui‐Ying Su, Xiao‐Yong Geng, Yang Yang, Hong‐Shan Yin

**Affiliations:** ^1^ Department of Cardiac Function Inspection The Third Hospital of Hebei Medical University Shijiazhuang China; ^2^ Department of Cardiology The Third Hospital of Hebei Medical University Shijiazhuang China

**Keywords:** Akt/ERK pathway, endoplasmic reticulum stress, myocardial ischaemia/reperfusion injury, NUCB2/nesfatin‐1

## Abstract

Nesfatin‐1 (encoded by NUCB2) is a cardiac peptide possessing protective activities against myocardial ischaemia/reperfusion (MI/R) injury. However, the regulation of NUCB2/nesfatin‐1 and the molecular mechanisms underlying its roles in MI/R injury are not clear. Here, by investigating a mouse MI/R injury model developed with transient myocardial ischaemia followed by reperfusion, we found that the levels of NUCB2 transcript and nesfatin‐1 amount in the heart were both decreased, suggesting a transcriptional repression of NUCB2/nesfatin‐1 in response to MI/R injury. Moreover, cardiac nesfatin‐1 restoration reduced infarct size, troponin T (cTnT) level and myocardial apoptosis, supporting its cardioprotection against MI/R injury in vivo. Mechanistically, the Akt/ERK pathway was activated, and in contrast, endoplasmic reticulum (ER) stress was attenuated by nesfatin‐1 following MI/R injury. In an in vitro system, similar results were obtained in nesfatin‐1‐treated H9c2 cardiomyocytes with hypoxia/reoxygenation (H/R) injury. More importantly, the treatment of wortmannin, an inhibitor of Akt/ERK pathway, abrogated nesfatin‐1 effects on attenuating ER stress and H/R injury in H9c2 cells. Furthermore, nesfatin‐1‐mediated protection against H/R injury also vanished in the presence of tunicamycin (TM), an ER stress inducer. Lastly, Akt/ERK inhibition reversed nesfatin‐1 effects on mouse ER stress and MI/R injury in vivo. Taken together, these findings demonstrate that NUCB2/nesfatin‐1 inhibits MI/R injury through attenuating ER stress, which relies on Akt/ERK pathway activation. Hence, our study provides a molecular basis for understanding how NUCB2/nesfatin‐1 reduces MI/R injury.

## 1 INTRODUCTION

Myocardial ischaemia/reperfusion (MI/R) injury is an unsolved medical issue that is caused by additional injuries derived from reperfusion therapy for patients with acute myocardial infarction (MI),^1^ one of the leading causes of morbidity and mortality in the world.^2^ Accumulating evidence has indicated that several aetiological factors, such as superfluous production of reactive oxygen species (ROS), disruption of calcium homoeostasis, and mitochondrial dysfunction, are major participators responsible for reperfusion‐mediated cytotoxicity, which eventually leads to contractile dysfunction and myocardial cell death.^3^, ^4^, ^5^ Despite of mechanistic discoveries, the successful development of therapeutic strategies targeting these pathological processes is still lacking, and no effective therapies are currently available to prevent or reduce MI/R injury, therefore creating an urgent need to resort to alternative approaches.^6^, ^7^, ^8^

In recent years, nesfatin‐1, a newly identified cardiac nucleobindin‐2 (NUCB2)‐derived hypothalamic peptide, has been shown to exhibit protective activities against MI/R injury, as indicated by reduced infarct size and lactate dehydrogenase (LDH) release, as well as decreased postischemic contracture.^9^ Interestingly, it was further demonstrated that these activates of nesfatin‐1 appear to involve multiple pro‐survival kinases including ERK1/2.^9^ Moreover, aside from this in vitro study, in an experimental isoproterenol‐induced MI rat model, nesfatin‐1 administration ameliorates myocardial injury, in which the inhibited apoptosis and inflammation by Akt/GSK‐3β signalling pathway is thought to be a potential mechanism.^10^ Together, these findings suggest a promising application of nesfatin‐1 to interfere MI/R injury. However, as far as we can know, the following critical issues including the regulation of NUCB2/nesfatin‐1 following MI/R injury, the in vivo evidence of nesfatin‐1 protection against MI/R injury, and its underlying molecular mechanisms have not been clarified.

In the present study, we show that nesfatin‐1 level in the heart is decreased following MI/R injury, which could be explained by simultaneous down‐regulation of NUCB2 transcription. We also reveal that cardiac nesfatin‐1 restoration reduces MI/R injury in vivo, and demonstrate that Akt/ERK pathway‐dependent attenuation of endoplasmic reticulum (ER) stress is responsible for this function of nesfatin‐1.

## 2 MATERIALS AND METHODS

### 2.1 MI/R injury model

Experimental MI/R model was established in C57BL/6 mice (male, 10‐12 weeks of age) through 30‐minute transient myocardial ischaemia followed by 4‐hour reperfusion as previously described.^11^ Briefly, mice were subjected to a left lateral thoracotomy and pericardiectomy, followed by the occlusion of left anterior descending coronary artery for 30 minutes, and then reperfused for 4 hours. Control mice received sham surgery performed with the same procedures except coronary artery ligation. Seven mice were randomly assigned into each group. For restoring nesfatin‐1 in the heart, mice were administrated intramyocardially with 5 or 10 ng/g bodyweight nesfatin‐1 at 30 minutes after ischaemia. For inhibiting Akt/ERK pathway, 1 μg/g bodyweight wortmannin was synchronously administrated intramyocardially with nesfatin‐1 at 30 minutes after ischaemia. The experimental protocols conformed to the Guidelines for the Care and Use of Laboratory Animals, and this study was approved by the Animal Care and Use Committee of The Third Hospital of Hebei Medical University.

### 2.2 Cell culture and H/R injury model

H9c2 cardiomyocyte cells were cultured, and H/R injury was induced following previous protocols.^12^ H9c2 cells were cultured in Dulbecco's modified Eagle's medium (DMEM) (Sigma‐Aldrich) containing 10% heat‐inactivated foetal bovine serum (Invitrogen) and 2 mmol/L glutamine (Invitrogen) in a humidified atmosphere with 5% CO_2_ at 37°C. To induce H/R injury in vitro, cells were cultured in DMEM without serum and glucose, and transferred to a hypoxic chamber with a humidified atmosphere of 0.1% O_2_, 5% CO_2_, and 95% N_2_ at 37°C. Following 7 hours of hypoxia, cells were re‐oxygenated for 2 hours under a normoxic condition in complete DMEM. Cells cultured with complete DMEM and under normoxic conditions throughout were prepared as counterparts.

### 2.3 qRT‐PCR analysis

Total RNA from heart tissues and cell samples was extracted by TRIzol Reagent kit (Invitrogen) according to the manufacturer's instructions. The complementary cDNA was synthesized from isolated RNA using the First‐Strand cDNA Synthesis Kit (Invitrogen). Then, qRT‐PCR analysis was performed on a CFX96 real‐time PCR system (Bio‐Rad) by using specific primers (available upon request) and SYBR‐green PCR master mix (Applied Biosystems) based on the manufacturer's protocols. The expression levels were analysed using 2^−ΔΔ^ *^Ct^* method.^13^

### 2.4 Western blotting

Total protein was extracted from heart tissues and cell samples using ice‐cold RIPA lysis buffer (Cell Signaling Technology). Denatured protein samples were loaded (30 μg for each well) onto 12% sodium dodecylsulfate‐polyacrylamide electrophoretic gels and separated, and then transferred to polyvinylidene difluoride (PVDF) membranes (Millipore). The PVDF membranes were immunoblotted with primary antibodies against Nesfatin‐1 (1:500, R&D Systems), β‐actin (1:5000, Santa Cruz Biotechnology), p‐Akt (1:2000, Abcam), Akt (1:2000, Abcam), p‐ERK (1:1000, Santa Cruz Biotechnology), ERK (1:1000, Santa Cruz Biotechnology), CHOP (1:500, Cell Signaling Technology), ATF6 (1:500, Proteintech), GPR78 (1:1000, Novus Biologicals) and caspase‐12 (1:1000, Santa Cruz Biotechnology), respectively. Immunoreactivity was determined with enhanced chemiluminescence (ECL) method and quantified by Image J software (version 1.47, NIH, Bethesda, MD, USA).

### 2.5 ELISA assay

The levels of mouse troponin T (CSB‐EL024016MO, CUSABIO) and nesfatin‐1 (NBP2‐81187, Novus Biologicals) were measured by ELISA assay according to the manufacturer's protocols. The activity of caspase‐3 was detected by Caspase‐3 Activity Assay Kit (#5723, Cell Signaling Technology), and the LDH activity was assessed by the Rat L‐lactate Dehydrogenase (L‐LDH) ELISA Kit (CSB‐E11324r, CUSABIO) following manufacturer's instructions. Optical density values were determined via a microplate reader (Bio‐Rad Laboratories).

### 2.6 Infarct size evaluation

The hearts were removed and cut into 2‐ to 3‐mm thickness sections, which were incubated in 0.1 mol/L sodium phosphate buffer containing 1% 2,3,5‐triphenyl tetrazolium chloride (TTC) (Sigma‐Aldrich) for 15 minutes at 37°C. Then, sections were fixed overnight in 4% formalin solution at 4°C. Afterwards, sections were photographed, and the infarct size (white area) was calculated using ImageJ software and expressed as a percentage of total heart size.

### 2.7 Statistical analysis

All values are expressed as mean ± standard deviation (SD). Statistics were performed by using GraphPad Prism Software version 5.0 with the Student's *t* test or one‐way ANOVA test. *P* < .05 was considered statistically significant.

## 3 RESULTS

### 3.1 Levels of NUCB2 transcript and nesfatin‐1 are decreased in the heart following MI/R injury

**FIGURE 1:**
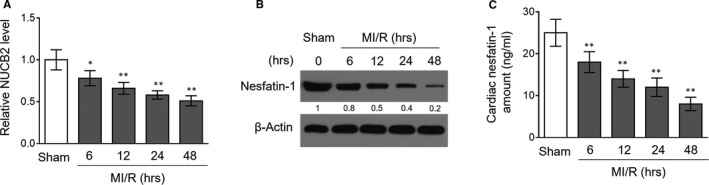
NUCB2/nesfatin‐1 is decreased in mouse heart following MI/R injury. (A‐C) NUCB2 transcript level (A), nesfatin‐1 protein expression (B), and nesfatin‐1 amount (C) in the homogenates of mouse heart following MI/R injury at different time periods as indicated. Mice with sham surgery were used as controls. Each group contains seven mice. Data are mean ± SD (n = 7). One‐way ANOVA test. *, *P* < .05; **, *P* < .01, vs sham group

Nesfatin‐1 alleviates MI/R injury in isolated and Langendorff‐perfused rat heart preparations,^9^ whereas how nesfatin‐1 level is regulated following MI/R injury in vivo is unclear. To address this issue, we utilized a mouse MI/R injury model induced with transient myocardial ischaemia followed by reperfusion.^14^ As nesfatin‐1 is encoded by NUCB2,^15^ we firstly evaluated NUCB2 transcript level in the heart of mouse with experimental MI/R injury. The quantitative reverse transcription‐PCR (qRT‐PCR) analysis showed that NUCB2 level in the heart was decreased time‐dependently following MI/R injury (Figure 1A), suggesting a down‐regulated NUCB2 transcription in this pathogenic condition. Next, we asked whether the encoded nesfatin‐1 is also decreased in the heart with MI/R injury. As determined by Western blotting analysis, the cardiac nesfatin‐1 level was indeed decreased similarly following MI/R injury compared with sham treatment (Figure 1B). In addition, to confirm this result, we directly assessed nesfatin‐1 amount in heart samples by applying enzyme‐linked immunosorbent assay (ELISA). In concert, the data showed that nesfatin‐1 level was lowered in mouse heart following MI/R injury (Figure 1C). Together, these results indicate that NUCB2 transcription is repressed along with corresponding decreased nesfatin‐1 expression in the heart subjected to MI/R injury.

### 3.2 Intramyocardial administration of nesfatin‐1 inhibits MI/R injury

**FIGURE 2:**
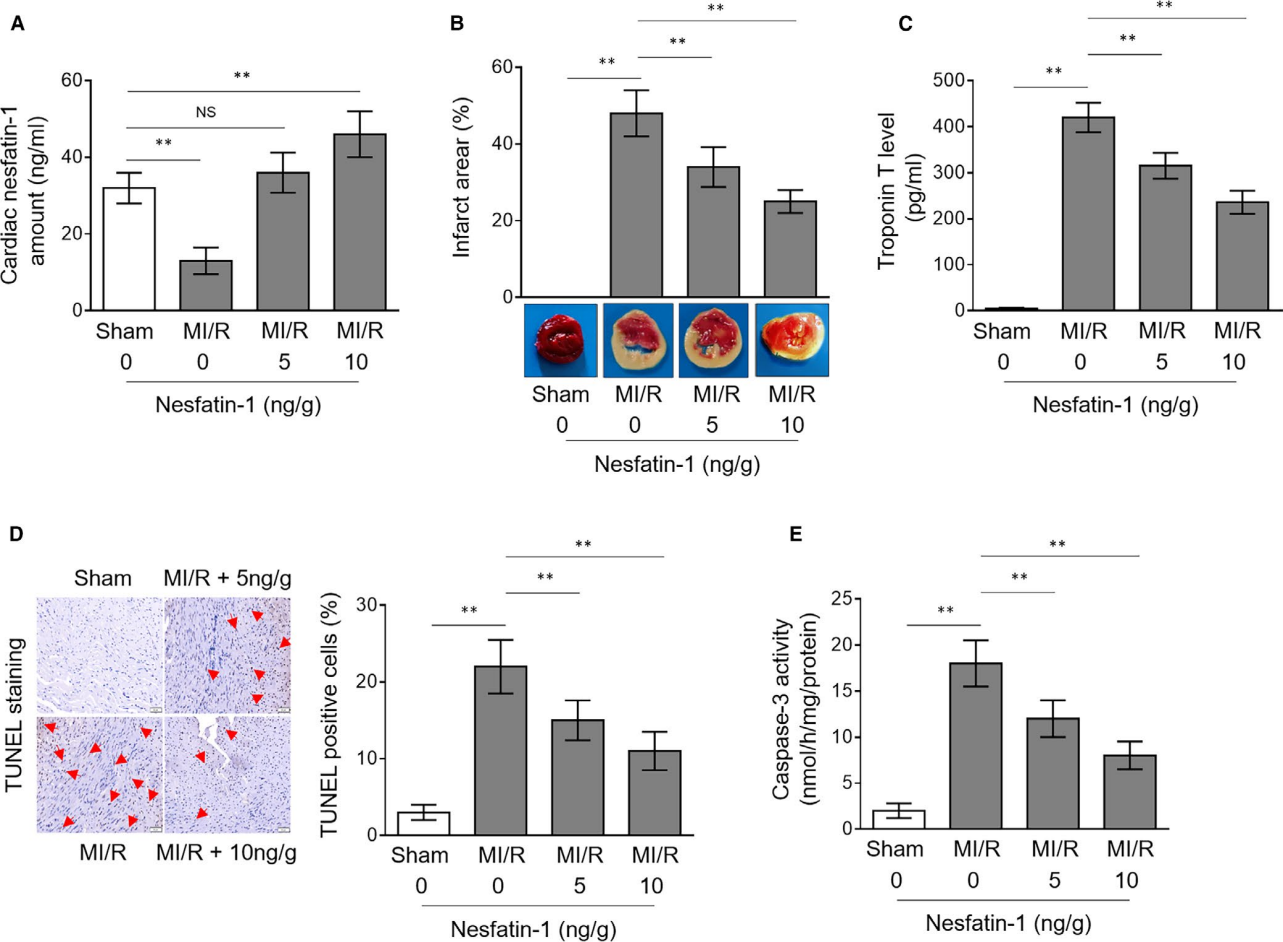
Nesfatin‐1 restoration ameliorates MI/R injury. (A‐E) Mice were intramyocardially administrated with 5 or 10 ng/g bodyweight nesfatin‐1 at 30 min after ischaemia. Each group contains 7 mice. (A) Nesfatin‐1 peptide amount in heart homogenates was measured by ELISA assay. (B) Infarct area in the heart slices was evaluated by TTC staining. (C) The cardiac troponin T level in the serum was assessed using ELISA assay. (D) Myocardial apoptosis was detected by TUNEL staining, and the percentage of apoptotic myocardial cells is depicted on the right. (E) The caspase‐3 activity in heart homogenates was determined by caspase activity assay. Data are mean ± SD (n = 7). Student's *t* test. *, *P* < .05; **, *P* < .01. NS, not significant

In previous studies, nesfatin‐1 shows cardioprotective effects on isoproterenol‐induced myocardial infarction and cerebral ischaemia‐reperfusion injury in rats.^10^, ^16^ However, whether nesfatin‐1 prevents MI/R injury in animal model is uncertain. As the cardiac nesfatin‐1 level is naturally decreased in mouse heart following MI/R injury, we attempted to examine the roles of nesfatin‐1 in this process via artificially restoring its level in the heart through intramyocardial injection. Two doses of nesfatin‐1 (5 and 10 ng/g bodyweight) were administrated in mice with MI/R injury, and both of them effectively restored nesfatin‐1 level to the level of sham group mice, as assessed by ELISA assay (Figure 2A). Consequently, the staining of heart slices with 2,3,5‐triphenyl tetrazolium chloride (TTC) showed that the infarct area in mouse heart with MI/R injury was obviously reduced when nesfatin‐1 was restored (Figure 2B). In agreement with this, the level of cardiac troponin T in the serum, a reliable diagnostic marker with high specificity to assess acute cardiac injury,^17^ was also decreased upon nesfatin‐1 restoration (Figure 2C). The elevated apoptosis of cardiomyocytes is a pivotal pathogenic factor for aggravating MI/R injury.^18^ Using the terminal deoxyribonucleotidyl transferase (TDT)‐mediated dUTP‐digoxigenin nick‐end labelling (TUNEL) assay to detect apoptosis in situ with heart slices, we found that nesfatin‐1 administration decreased the population of apoptotic cardiomyocytes (Figure 2D), which is further confirmed by decreased caspase‐3 activity in heart homogenates, as determined by the caspase activity assay (Figure 2E). These data suggest that nesfatin‐1‐inhibited apoptosis is at least one of the mechanisms through which nesfatin‐1 protects against MI/R injury in vivo.

### 3.3 Nesfatin‐1 activates Akt/ERK pathway and attenuates ER stress

**FIGURE 3:**
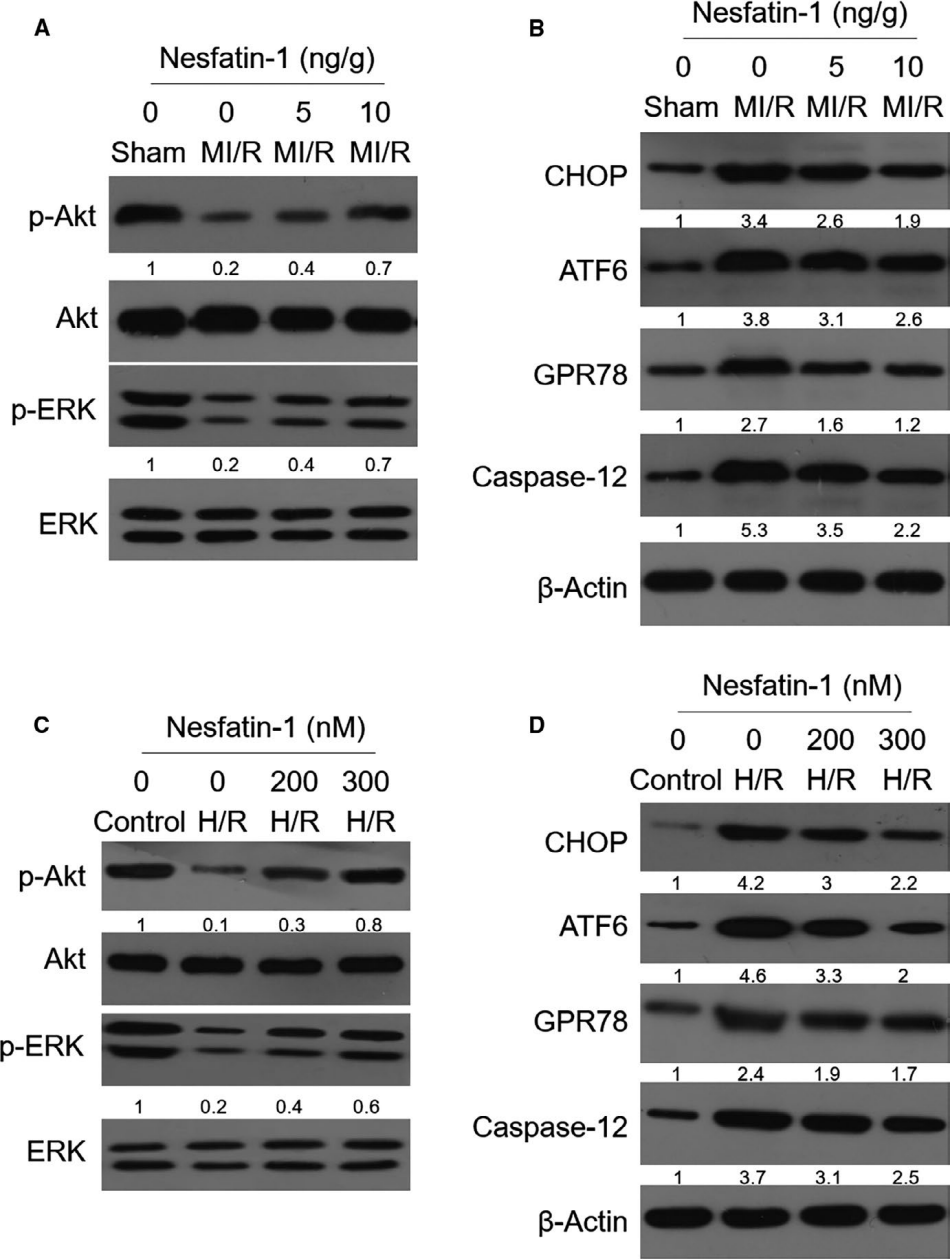
Nesfatin‐1 activates Akt/ERK pathway and attenuates ER stress. (A‐B) Mice were treated as in Figure 2. The protein expression of p‐Akt, Akt, p‐ERK, and ERK (A) and CHOP, ATF6, GPR78, and caspase‐12 (B) in heart homogenates was determined by Western blotting analysis. Images are representative of 3 independent experiments. (C‐D) H9c2 cells cultured in vitro were subjected to control treatment or hypoxia/reoxygenation (H/R) injury for 24 h with or without the addition of 200 nmol/L or 300 nmol/L nesfatin‐1 as indicated. The protein expression of p‐Akt, Akt, p‐ERK, and ERK (C) and CHOP, ATF6, GPR78, and caspase‐12 (D) was determined by Western blotting analysis. Images are representative of 3 independent experiments

The Akt/ERK pathway is mechanistically involved in the pathogenesis of MI/R injury.^19^, ^20^ Besides, nesfatin‐1 has been shown to activate Akt/ERK pathway in rat cardiomyocytes.^21^ To seek the mechanisms of nesfatin‐1 protection against MI/R injury, we checked the activation status of Akt/ERK pathway in the heart samples of mice treated with nesfatin‐1. Western blotting analysis revealed that the levels of phosphorylation of Akt and ERK were rescued by nesfatin‐1 to a significant extent in the heart of mouse with MI/R injury, but the basal levels of Akt and ERK were not affected obviously (Figure 3A), therefore describing that the activation of Akt/ERK pathway is recovered by nesfatin‐1. A previous study demonstrates that the Akt/ERK pathway has critical role in counteracting ER stress‐induced cell death,^22^ a mechanism leading to the induction of cardiomyocyte apoptosis following MI/R injury.^23^, ^24^ By analysing the levels of ER stress markers in heart samples, we noticed that along with the activation of Akt/ERK pathway, ER stress synchronously declined in response to nesfatin‐1 administration, as evidenced by decreased protein levels of CHOP, ATF6, GPR78 and caspase‐12 (Figure 3B). Collectively, these results suggest that the restoration of nesfatin‐1 in the heart results in Akt/ERK pathway activation and ER stress attenuation following MI/R injury, which possibly underlie nesfatin‐1 protective effects against cardiomyocyte apoptosis and MI/R injury.

Next, in an attempt to extend our findings, we further applied an in vitro model to mimic MI/R injury, in which H9c2 cells were subjected to hypoxia/reoxygenation (H/R) injury.^25^ In consistence, we found that the repressed status of Akt/ERK pathway in H9c2 cells with H/R injury was reversed to a large extent when treated with exogenous nesfatin‐1 (Figure 3C). And furthermore, similar to phenotypes observed in mouse heart samples, compared with control treatment, the ER stress was augmented in H9c2 cells subjected to H/R injury, which was prominently suppressed in nesfatin‐1‐treated group, as indicated by changes of ER stress markers (Figure 3D). Thus, nesfatin‐1 is also able to activate Akt/ERK pathway and, meanwhile, suppress ER stress in H9c2 cells in vitro, mimicking those findings obtained in MI/R injury mouse model.

### 3.4 Akt/ERK inhibition reverses nesfatin‐1‐reduced ER stress and protection against H/R injury in H9c2 cells

**FIGURE 4:**
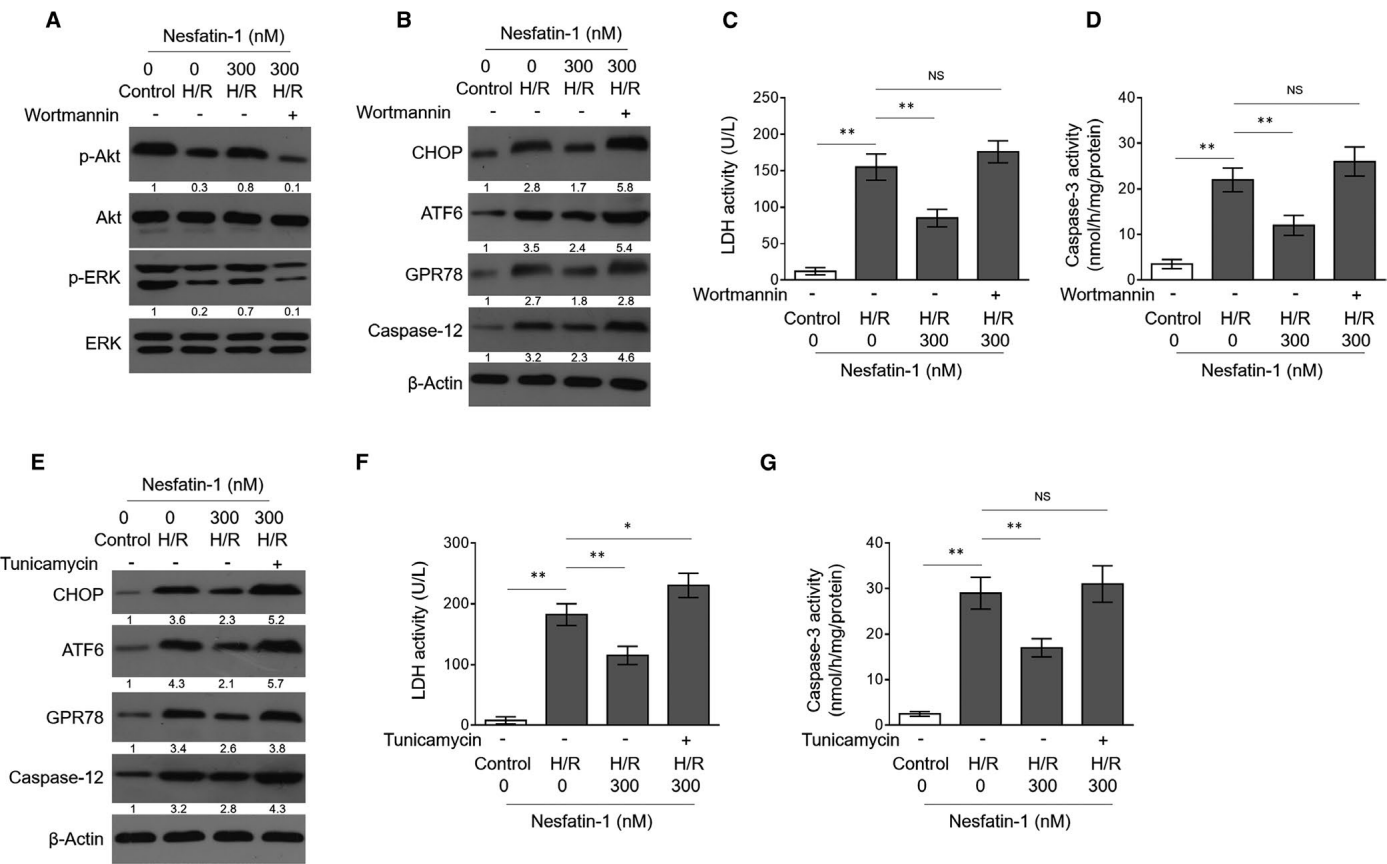
Akt/ERK inhibition abrogates nesfatin‐1 protection against H/R injury in H9c2 cells by inducing ER stress. (A) H9c2 cells were subjected to control treatment or H/R injury for 24 h with or without the addition of 300 nmol/L nesfatin‐1 and 100 nmol/L wortmannin as indicated. The protein expression of p‐Akt, Akt, p‐ERK, and ERK (A) and CHOP, ATF6, GPR78, and caspase‐12 (B) was determined by Western blotting analysis. Images are representative of three independent experiments. (C) LDH activity in the culture medium was spectrophotometrically assessed by LDH assay kit. (D) The caspase‐3 activity in H9c2 homogenates was determined by caspase activity assay. (E‐G) H9c2 cells were subjected to control treatment or H/R injury for 24 h with or without the addition of 300 nmol/L nesfatin‐1 and 10 μg/mL tunicamycin as indicated. (E) The protein expression of CHOP, ATF6, GPR78 and caspase‐12 was determined by Western blotting analysis. Images are representative of three independent experiments. (F‐G) LDH activity in culture medium (F) and caspase‐3 activity in H9c2 homogenates (G) were determined. Data are mean ± SD (3). Student's *t* test. *, *P* < .05; **, *P* < .01; NS, not significant

To gain a further mechanistic insight into the role of Akt/ERK pathway activation and ER stress attenuation in nesfatin‐1‐treated H9c2 cells with H/R injury, we inhibited Akt kinase activation with wortmannin, a pharmacological inhibitor of PI3K/Akt.^26^ As expected, wortmannin addition abrogated Akt/ERK pathway activation by nesfatin‐1 in H9c2 cells with H/R injury (Figure 4A). Keeping pace with this, wortmannin overtly induced ER stress in nesfatin‐1‐treated H9c2 cells with H/R injury (Figure 4B), hence establishing a causal link between Akt/ERK pathway activation and ER stress attenuation elicited by nesfatin‐1 under this condition. Functionally, upon wortmannin treatment, nesfatin‐1‐alleviated H/R injury in H9c2 cells vanished, as presented by increased LDH release (Figure 4C) and caspase‐3 activity (Figure 4D). Hence, these results prove that nesfatin‐1 reduces H/R injury through Akt/ERK pathway‐mediated ER stress inhibition. To further consolidate this conclusion, we induced ER stress pharmacologically in nesfatin‐1‐treated H9c2 cells with tunicamycin^27^ (Figure 4E). Consistently, similar to wortmannin treatment, nesfatin‐1‐restricted H/R injury in H9c2 cells was diminished in the presence of tunicamycin (Figure 4F,G). Altogether, these lines of evidence demonstrate that the protective roles of nesfatin‐1 against H/R injury rely on attenuating ER stress via Akt/ERK pathway activation.

### 3.5 Akt/ERK inhibition abrogates nesfatin‐1 effects on mouse ER stress and MI/R injury

**FIGURE 5:**
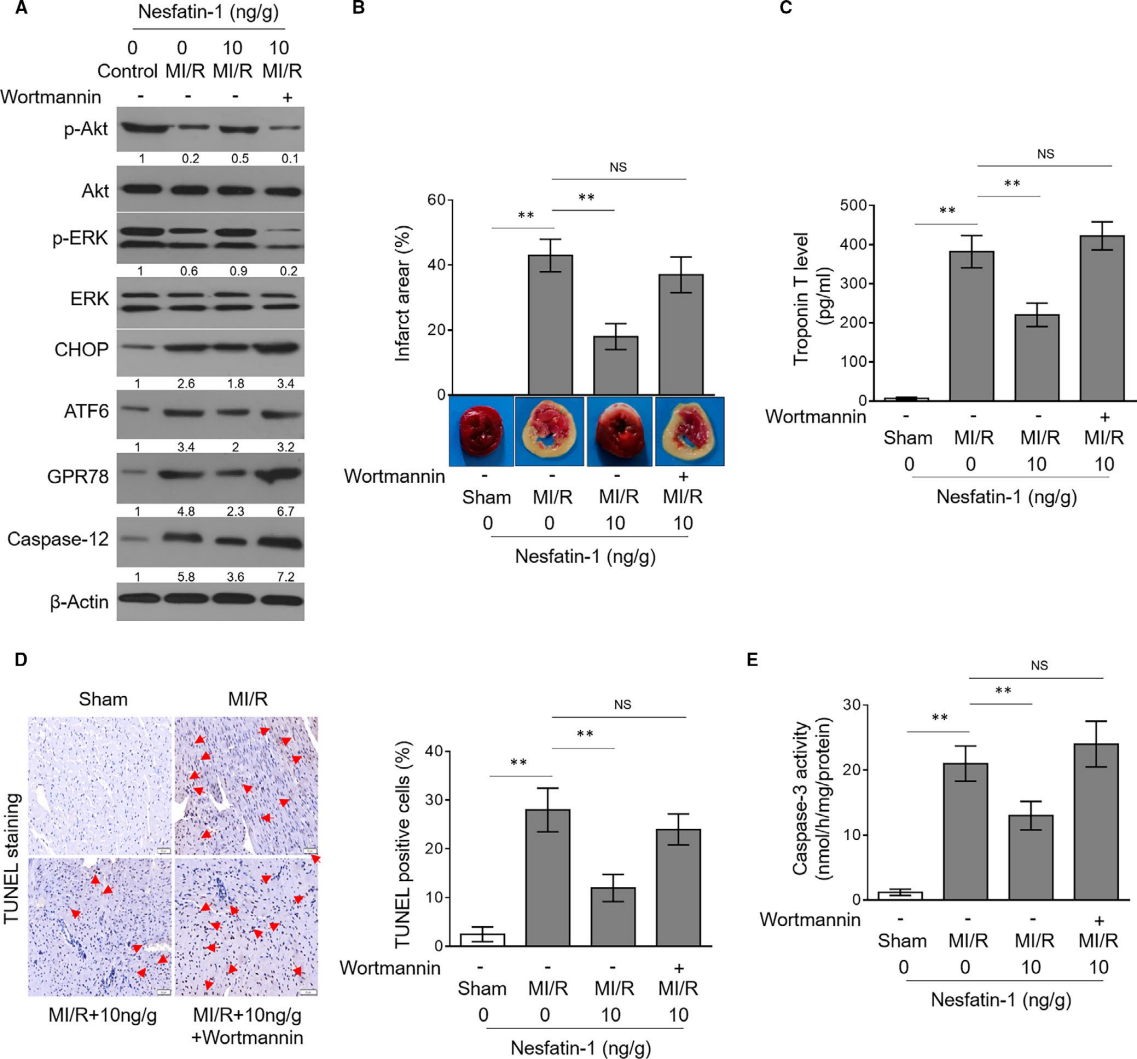
Akt/ERK inhibition significantly reverses nesfatin‐1 activities of reducing ER stress and attenuating MI/R injury. (A‐E) Mice were administrated intramyocardially with 10 ng/g bodyweight nesfatin‐1 combined with or without 1 μg/g bodyweight wortmannin at 30 min after ischaemia. Each group contains seven mice. (A) The protein expression of p‐Akt, Akt, p‐ERK, ERK, CHOP, ATF6, GPR78 and caspase‐12 in heart homogenates was determined by Western blotting analysis. Images are representative of three independent experiments. (B) Infarct area in the heart slices was evaluated by TTC staining. (C) Plasma cardiac troponin T level in the serum was assessed using ELISA assay. (D) Myocardial apoptosis was detected by TUNEL staining, and the percentage of apoptotic myocardial cells is depicted on the right. (E) The caspase‐3 activity in heart homogenates was determined by caspase activity assay. Data are mean ± SD (7). Student's *t* test. *, *P* < .05; **, *P* < .01; NS, not significant

Lastly, we asked whether inhibiting Akt/ERK pathway could also reverse nesfatin‐1 effects on ER stress and MI/R injury in vivo. To this end, wortmannin was administrated intramyocardially along with nesfatin‐1 into mice with MI/R injury. The inhibitory effect of wortmannin on Akt/ERK pathway was validated by determining the phosphorylated and basal levels of Akt and ERK in heart homogenates (Figure 5A). Consequently, similar to in vitro results, nesfatin‐1‐relieved ER stress was resurged by wortmannin following MI/R injury (Figure 5A), illustrating that nesfatin‐1 constrains ER stress via activating Akt/ERK pathway in vivo. To further establish a possible functional connection between this mechanism and nesfatin‐1 effects on MI/R injury, we evaluated the degree of MI/R injury when Akt/ERK pathway was inhibited by wortmannin. TTC staining of heart slices showed that the reduced infarct area (Figure 5B) and decreased cardiac troponin T (Figure 5C) by nesfatin‐1 in mice with MI/R injury was abrogated upon wortmannin combination administration. Furthermore, in line with the changes of MI/R injury, TUNEL assay also depicted that wortmannin treatment recovered the number of apoptotic cardiomyocytes, which would otherwise decline if nesfatin‐1 was administrated alone (Figure 5D). The impact of wortmannin on cardiomyocyte apoptosis following MI/R injury was reinforced by the re‐emerged increase in caspase‐3 activity (Figure 5E), keeping in concert with its effect on MI/R injury. In sum, these lines of evidence highlight a critical role of Akt/ERK pathway‐dependent attenuation of ER stress in mediating the protection of nesfatin‐1 against MI/R injury.

## 4 DISCUSSION

MI/R injury remains an intractable situation that may cause disability and mortality in AMI patients. Despite the current dilemma to effectively treat MI/R injury, a number of studies investigating multiple animal MI/R injury models have suggested that some bioactive peptides could display protective activities against MI/R injury, such as vasonatrin,^28^ circulating ghrelin,^29^ intermedin^30^ and recombinant human brain natriuretic peptide.^31^ These findings attract much research interest as bioactive peptides have several advantages to be candidate drugs for treating MI/R injury, including hypotoxicity and low immunogenicity, and favourable solubility and pharmacokinetic profile.^32^ Apart from the above‐mentioned peptides, nesfatin‐1, a well‐established anorexigenic peptide (82 amino acids) that is widely expressed at both central and peripheral tissues with pleiotropic effects on reducing food intake and bodyweight,^33^, ^34^ has been implicated in cardiovascular pathophysiology,^35^ and also shown to protect against I/R injury.^9^, ^16^ In the present study, we investigated this effect of nesfatin‐1 with both in vivo and in vitro MI/R injury models and further explored its regulation and the underlying mechanisms. We found that nesfatin‐1 was decreased in mouse models following MI/R injury, and instead, nesfatin‐1 restoration inhibited MI/R injury, hence providing in vivo evidence verifying the beneficial role of nesfatin‐1 against MI/R injury. We further demonstrate that this effect of nesfatin‐1 is dependent on attenuating ER stress via activating the signalling pathway of Akt/ERK, based on the observations that Akt/ERK pathway inhibition or ER stress induction abrogates nesfatin‐1 protection against MI/R injury. In summary, our findings underscore a pivotal role of Akt/ERK pathway‐regulated ER stress in the pathogenesis of MI/R injury and also offer a theoretical foundation to the promising application of nesfatin‐1 in future treatment of MI/R injury.

Nesfatin‐1 is encoded by NUCB2. We found that both levels of them were decreased in mouse heart samples following MI/R injury. Previous study has reported that the plasma nesfatin‐1 levels were decreased in AMI patients.^36^ An association between lower plasma nesfatin‐1 levels and SYNTAX score was also found in patients with non‐ST segment elevation myocardial infarction.^37^ It therefore would be of great clinical value to study whether nesfatin‐1 also declines in the plasma of AMI patients following MI/R injury, and further determine whether nesfatin‐1 could be exploited as a feasible diagnostic molecule. On the other hand, regardless of the complicated tissue resources of nesfatin‐1 secretion,^38^ the synchronized down‐regulation of NUCB2 and nesfatin‐1 in heart samples at least partly suggests a depressed transcription in response to MI/R injury. As revealed by our data, since MI/R injury is accompanied by nesfatin‐1 decrease and, oppositely, its exogenous anaplerosis alleviates the severity of MI/R injury, it turns to be important for future studies to investigate how NUCB2 is transcriptionally suppressed under this pathogenic condition. As MI/R injury is more severe when reperfusion is prolonged if the duration of ischaemia is equal, the observed time‐dependently decreased levels of NUCB2 transcript and nesfatin‐1 expression strongly suggest that they may be inversely correlated with cardiac injury in the heart subjected to transient myocardial ischaemia followed by reperfusion. More direct evidence is needed to show how NUCB2 transcription is associated with the severity of MI/R injury. Addressing the above issues will be helpful for us to understand how to maintain the cardiac haemostasis of nesfatin‐1 in order to interfere the pathogenic process of MI/R injury.

Since its discovery in 2006, we have witnessed an immense increase in the knowledge of multitude functions of nesfatin‐1, like those in glucose and lipid metabolism, blood pressure, and other activities in cardiovascular system.^39^, ^40^ The cardioprotective effects of nesfatin‐1 were first described in an ex vivo experiment using the Langendorff‐perfused rat heart preparations.^9^ But, paradoxically, another study reported that in isolated neonatal rat hearts, nesfatin‐1 could induce cardiomyocyte apoptosis.^41^ The clarity of this discrepancy is impeded due to the lack of direct in vivo evidence. In this study, we addressed this issue by treating MI/R injured mice with intramyocardial injection of nesfatin‐1. Subsequent evaluation of infarct area and cardiomyocyte apoptosis provides evidence demonstrating a protective role of nesfatin‐1 in this mouse MI/R injury model. In addition, we also observed that nesfatin‐1 protected against H/R injury in H9c2 cardiomyocytes in vitro. Although it is still uncertain whether similar conclusion could be drawn in other MI/R injury models, these lines of evidence undoubtedly support a positive role of nesfatin‐1 in reducing MI/R injury and cardiomyocyte apoptosis, at least in our experimental systems.

Studies have shown that the dysregulated ER stress is a vital aetiological factor for mediating MI/R injury, which is a target of bioactive peptides. For instance, inhibition of ER stress by intermedin protects against MI/R injury through a PI3K‐Akt signalling pathway.^42^ Moreover, inhibition of ER stress by ghrelin protects against MI/R injury in rats.^43^ Furthermore, vasonatrin protects the diabetic heart against ischaemia‐reperfusion injury by inhibiting ER stress.^28^ Except peptides, it has also been shown that bFGF protects against MI/R injury through attenuating ER stress and mitochondrial injury via PI3K/Akt/ERK1/2 pathway activation.^19^ Reportedly, NUCB2 plays an important role in adaptation to ER stress in melanoma cells.^44^ In our study, in addition to Akt/ERK pathway activation and concurrent ER stress attenuation observed upon nesfatin‐1 treatment following MI/R injury, similar results were obtained in H9c2 cells with H/R injury. However, when Akt/ERK pathway was inhibited, nesfatin‐1‐mediated ER stress attenuation and protection in H/R injured H9c2 cells and mouse MI/R injury vanished. Hence, in concert with previous literatures, our study mechanistically highlights an important role of Akt/ERK pathway‐attenuated ER stress in suppressing cardiomyocyte apoptosis and MI/R injury, which underlies nesfatin‐1‐mediated cardioprotective effects. Future studies are warranted to investigate the possibility of involving nesfatin‐1 in the therapy for MI/R injury. Further, in concert with our study, the positive effect of nesfatin‐1 on Akt phosphorylation has been reported in many circumstances,^10^, ^45^, ^46^ suggesting a ubiquitous activity of nesfatin‐1 on this signalling pathway. We speculate that the phosphoinositide 3‐kinase (PI3‐Kinase), an upstream regulator of Akt,^47^ may play an intermediate role in mediating nesfatin‐1 effect on Akt, as it has been related to the regulatory network of nesfatin‐1.^48^, ^49^ This clue could be helpful to address the mechanism of nesfatin‐1 in modulating Akt/ERK pathway.

## CONFLICT OF INTEREST

None.

## AUTHOR CONTRIBUTION

**Rui‐Ying Su:** Conceptualization (lead); Data curation (equal); Resources (equal); Supervision (equal); Writing‐original draft (equal); Writing‐review & editing (equal). **Xiao‐Yong Geng:** Data curation (equal); Resources (equal); Software (equal). **Yang Yang:** Formal analysis (equal); Investigation (equal); Software (equal). **Hong‐Shan Yin:** Methodology (equal); Validation (equal).
